# Oncologic outcomes of screen-detected and non-screen-detected T1 colorectal cancers

**DOI:** 10.1055/a-2263-2841

**Published:** 2024-03-13

**Authors:** Lisa van der Schee, Krijn J. C. Haasnoot, Sjoerd G. Elias, Kim M. Gijsbers, Yasser A. Alderlieste, Yara Backes, Anne-Marie van Berkel, Femke Boersma, Frank ter Borg, Emilie C.H. Breekveldt, Koen Kessels, Miriam Koopman, Iris Lansdorp-Vogelaar, Monique E. van Leerdam, Gertjan Rasschaert, Ramon-Michel Schreuder, Ruud W.M. Schrauwen, Tom C.J. Seerden, Marcel B.W. Spanier, Jochim S. Terhaar sive Droste, Esther Toes-Zoutendijk, Jurriaan B. Tuynman, Geraldine R. Vink, Wouter H. de Vos tot Nederveen Cappel, Frank P. Vleggaar, Miangela M. Laclé, Leon M. G. Moons

**Affiliations:** 1 8124 Gastroenterology and Hepatology, University Medical Centre Utrecht, Utrecht, Netherlands; 2 8124 Pathology, University Medical Centre Utrecht, Utrecht, Netherlands; 3 8124 Julius Center for Health Sciences and Primary Care, University Medical Centre Utrecht, Utrecht, Netherlands; 4 8124 Gastroenterology and Hepatology, University Medical Centre Utrecht, Utrecht, Netherlands; 5 2976 Gastroenterology and Hepatology, Deventer Hospital, Deventer, Netherlands; 6 159172 Gastroenterology and Hepatology, Beatrixziekenhuis, Gorinchem, Netherlands; 7 1140 Gastroenterology and Hepatology, Noordwest Ziekenhuisgroep, Alkmaar, Netherlands; 8 72485 Gastroenterology and Hepatology, Gelre Hospitals, Apeldoorn, Netherlands; 9 6993 Public Health, Erasmus MC, Rotterdam, Netherlands; 10 1228 Gastrointestinal Oncology, The Netherlands Cancer Institute - Antoni van Leeuwenhoek Hospital, Amsterdam, Netherlands; 11 6028 Gastroenterology and Hepatology, Sint Antonius Ziekenhuis, Nieuwegein, Netherlands; 12 8124 Medical Oncology, University Medical Centre Utrecht, Utrecht, Netherlands; 13 4501 Gastroenterology and Hepatology, Leiden University Medical Center, Leiden, Netherlands; 14 89411 Gastroenterology and Hepatology, Amphia Hospital, Breda, Netherlands; 15 3168 Gastroenterology and Hepatology, Catharina Hospital, Eindhoven, Netherlands; 16 97772 Gastroenterology and Hepatology, Bernhoven Hospital Location Uden, Uden, Netherlands; 17 1322 Gastroenterology and Hepatology, Rijnstate Hospital Arnhem Branch, Arnhem, Netherlands; 18 10233 Gastroenterology and Hepatology, Jeroen Bosch Hospital, Den Bosch, Netherlands; 19 522567 Surgery, Amsterdam University Medical Centres, Amsterdam, Netherlands; 20 Research and Development, Netherlands Comprehensive Cancer Organisation, Utrecht, Netherlands; 21 8772 Gastroenterology and Hepatology, Isala Hospital, Zwolle, Netherlands

## Abstract

**Background** The incidence of T1 colorectal cancer (CRC) has increased with the implementation of CRC screening programs. It is unknown whether the outcomes and risk models for T1 CRC based on non-screen-detected patients can be extrapolated to screen-detected T1 CRC. This study aimed to compare the stage distribution and oncologic outcomes of T1 CRC patients within and outside the screening program.

**Methods** Data from T1 CRC patients diagnosed between 2014 and 2017 were collected from 12 hospitals in the Netherlands. The presence of lymph node metastasis (LNM) at diagnosis was compared between screen-detected and non-screen-detected patients using multivariable logistic regression. Cox proportional hazard regression was used to analyze differences in the time to recurrence (TTR), metastasis-free survival (MFS), cancer-specific survival (CSS), and overall survival. Additionally, the performance of conventional risk factors for LNM was evaluated across the groups.

**Results** 1803 patients were included (1114 [62%] screen-detected), with median follow-up of 51 months (interquartile range 30). The proportion of LNM did not significantly differ between screen- and non-screen-detected patients (12.6% vs. 8.9%; odds ratio 1.41; 95%CI 0.89–2.23); a prediction model for LNM performed equally in both groups. The 3- and 5-year TTR, MFS, and CSS were similar for patients within and outside the screening program. However, overall survival was significantly longer in screen-detected T1 CRC patients (adjusted hazard ratio 0.51; 95%CI 0.38–0.68).

**Conclusions** Screen-detected and non-screen-detected T1 CRCs have similar stage distributions and oncologic outcomes and can therefore be treated equally. However, screen-detected T1 CRC patients exhibit a lower rate of non-CRC-related mortality, resulting in longer overall survival.

## Abstract (de)

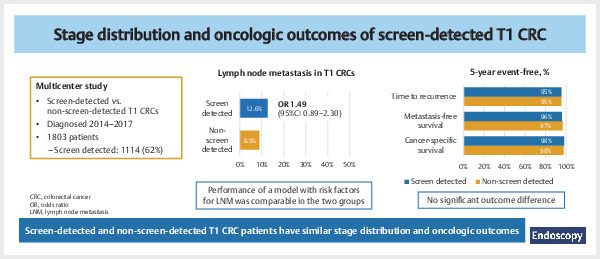

## Introduction

Population-based colorectal cancer (CRC) screening has led to a stage shift in the screen-detected CRCs, characterized by a higher proportion of stage I and a lower proportion of stage IV CRCs compared with non-screen-detected CRCs 1 . Whether this more favorable stage distribution is also observed in screen-detected submucosal invasive (T1) CRCs specifically is unknown. Given the increasing use of organ-preserving treatment strategies for T1 CRC, it is essential to determine whether predictors of metastasis and oncologic outcomes in screen-detected T1 CRCs are comparable with those in non-screen-detected cases. Especially as most risk prediction models used to date are based on data from non-screen-detected T1 CRCs from the prescreening period.

Based on the significant reduction in advanced-stage CRC incidence after the implementation of CRC screening programs, it might be assumed that stage migration owing to earlier detection also extends to screen-detected T1 CRCs, and that screen-detected T1 CRCs are detected at an earlier stage, before metastasis occurs. However, it cannot be definitely established that T-stage migration owing to screening consistently coincides with migration in terms of lymph node involvement (N stage) and distant metastasis (M stage), as recent genetic insights have suggested that the development of lymph node and distant metastases may be already determined in the earliest phases of cancer development 2 .

Up until now, only a limited number of small-scale studies have examined the risk of lymph node metastasis (LNM) or CRC recurrence specifically in screen-detected T1 CRC 3 4 5 6 , showing a lower or comparable risk of LNM and CRC recurrence compared with T1 CRCs from the pre-screening era. However, these studies either had a short follow-up, excluded primary surgical resections, or had small sample sizes with few events. Furthermore, none of the studies directly compared oncologic outcomes between screen-detected and non-screen-detected T1 CRC patients.

Therefore, the objectives of the current study were to evaluate the stage distribution and oncologic outcomes of screen-detected and non-screen-detected T1 CRC patients, and to study whether the established risk factors for LNM, which are used to guide clinical decision-making, can be extrapolated to the screening population of T1 CRC patients.

## Methods

### Study design and selection of patients

A multicenter retrospective observational cohort study was conducted. All consecutive patients diagnosed with T1 CRC between 2014 and 2017 from 12 hospitals (including one academic hospital) were identified through the Netherlands Cancer Registry. The Dutch National screening program started in 2014, with a stepwise introduction by age cohorts, until all eligible age cohorts had been invited at least once in 2019. Men and women aged 55–75 years are invited once every 2 years to send in stool samples for detection of occult blood using a fecal immunochemical test (FIT). During our study period, the invitation coverage was 40% of the target population in 2014, rising to 95% in 2017 1 .

The reason for index colonoscopy (i.e. population-based CRC screening program or other reason) was retrieved from the individual electronic medical records, which were reviewed at the participating hospitals. Patients were included when the local pathology report confirmed the diagnosis of pT1 CRC. Exclusion criteria were: (i) hereditary predisposition to CRC (Lynch or familial adenomatous polyposis syndrome); (ii) inflammatory bowel disease; (iii) synchronous CRC at diagnosis or previous CRC ≤5 years before the diagnosis of the T1 CRC; (iv) non-adenocarcinoma; (v) missing pathology or endoscopy reports; and (vi) neoadjuvant (chemo-)radiotherapy. Further details on the data collection and definitions of study parameters can be found in **Appendix 1s** , see online-only Supplementary material.

This study was exempt from institutional board review by the Medical Ethical Review Committee of the University Medical Center Utrecht (reference number 15–487/C) and was carried out in accordance with the Helsinki Declaration. The study confirmed to the STROBE (Strengthening the Reporting of Observational Studies in Epidemiology) guideline for cohort studies.

### Determinant and end points

The determinant of interest was the indication for colonoscopy (i.e. screen detected or non-screen detected). Screen-detected T1 CRC was defined as T1 CRC detected through the population-based screening program after a positive FIT. All clinically detected T1 CRCs outside the screening program were considered to be non-screen detected.

The primary outcome was the proportion of synchronous LNM. This was defined as tumor-positive lymph nodes in the surgical resection specimen at the time of diagnosis. In addition, we studied whether the performance of conventional clinical and histopathologic risk factors for LNM was similar in screen-detected and non-screen-detected patients.

Secondary outcomes were 3- and 5-year time to recurrence (TTR), metastasis-free survival (MFS), colorectal cancer-specific survival (CSS), and overall survival. TTR was defined as the time from diagnosis until the event of CRC recurrence (locoregional and/or distant recurrence) or CRC-related death. MFS was defined as the time from diagnosis until the event of distant recurrence (i.e. outside the area intended to be removed with salvage surgery) or CRC-related death. CSS was defined as the time from diagnosis until the event of CRC-related or CRC treatment-related death. Overall survival was defined as the time from diagnosis until the event of death due to any cause.

### Data analysis

Baseline characteristics were compared between screen-detected and non-screen-detected T1 CRC patients using the chi-squared test for categorical variables and the Mann–Whitney *U* test for continuous data. Categorical data were expressed as frequencies with percentages, and continuous data as the median with corresponding interquartile range (IQR).

We evaluated the potential association between the indication (screen detected or non-screen detected) and LNM with univariable and multivariable logistic regression analyses in the group of surgically treated patients (both primary and completion surgery). In the multivariable model, we adjusted for age, sex, polyp location, polyp morphology, polyp size, differentiation grade, lymphovascular invasion (LVI), and the number of lymph nodes retrieved during surgery. These factors were chosen as potential confounders based on the available literature. As a sensitivity analysis, we also studied the association between the indication for colonoscopy and LNM restricted to patients aged 55–80 years, which is the age range of the screening population. The upper age limit of 80 was chosen to take into account screen-detected patients aged >75 years at diagnosis owing to delays in screening invitation, returning the FIT, or CRC treatment.

To evaluate whether established conventional clinical and histologic risk factors for LNM are also predictive in screen-detected T1 CRC patients, we analyzed the performance of a logistic regression model based on these risk factors in the screen-detected and non-screen-detected patients separately. The risk factors included were age, location, morphology, differentiation grade, and LVI 7 8 9 10 . The main performance measures were the calibration and the discriminative ability of the model. Calibration was assessed graphically using smoothed calibration curves. Discriminative ability was quantified by the area under the receiver operating curve (AUROC). The discriminative performance of the model (AUROC) was compared between both groups using a *Z* test. Furthermore, a closed testing procedure was performed to assess whether the model, which was developed in the total cohort, needed updating in the screen-detected or non-screen-detected groups, or in both (e.g. re-estimation of the intercept and/or slope, or re-estimation of all coefficients).

The TTR, MFS, CSS, and overall survival of screen-detected and non-screen-detected patients were described using the Kaplan–Meier method. A Cox proportional hazard regression model was used to study the association between the indication for colonoscopy (i.e. screen detected or non-screen detected) and oncologic and survival outcomes, adjusted for potential confounders. For TTR, CSS, and MFS, we corrected for polyp location, treatment strategy, differentiation grade, LVI, and resection margin status. For overall survival, we adjusted for age at diagnosis, sex, and American Society of Anesthesiologists (ASA) classification. Again, we performed a sensitivity analysis in which we included only patients aged 55–80 years. Additionally, we repeated all analyses in a subgroup of patients who were treated first with endoscopic resection.

Because of missing data for several clinicopathologic variables ( Table 1 ), we performed multiple imputation before data analysis (using multivariate imputation by chained equations with 22 variables, 10 imputation data sets, and 21 iterations). Rubin’s rules were used to pool results across imputed datasets. All statistical analyses were performed using R version 4.0.3 (RStudio, Inc., Boston, Massachusetts, USA). A two-sided *P* value ≤0.05 was considered significant.

**Table:** **Table 1** Clinical and histopathologic characteristics of the 1803 included T1 colorectal cancer (CRC) patients.

	Baseline characteristics			*P* value*
	Total cohort (n = 1803)	T1 CRC patients		
		Screen detected (n = 1114)	Non-screen detected (n = 689)	
Age, median (IQR), years	69 (11)	67 (10)	71 (15)	<0.001
Sex, male, n (%)	1124 (62.3)	726 (65.2)	398 (57.7)	0.002
ASA classification, n (%)†				<0.001
I	424 (24.6)	277 (25.7)	147 (22.8)	
II	1067 (61.9)	702 (65.1)	365 (56.6)	
III–IV	233 (13.5)	100 (9.3)	133 (20.6)	
Missing	79	35	44	
Location, n (%)				<0.001
Right-sided colon	321 (17.8)	181 (16.2)	140 (20.3)	
Left-sided colon	1058 (58.7)	698 (62.7)	360 (52.2)	
Rectum	424 (23.5)	235 (21.1)	189 (27.4)	
Morphology, n (%)†				0.99
Nonpedunculated	1164 (65.8)	724 (65.9)	440 (65.7)	
Pedunculated	604 (34.2)	374 (34.1)	230 (34.3)	
Missing	35	16	19	
Polyp size, median (IQR)†				<0.001
Diameter, mm	20 (15)	20 (17)	20 (15)	
Missing	181	83	98	
Differentiation, n (%)†				0.99
Well/moderate	1693 (94.9)	1044 (94.8)	639 (95.0)	
Poor	91 (5.1)	57 (5.2)	34 (5.0)	
Missing	19	13	6	
Lymphovascular invasion, n (%)†				0.72
Absent	1470 (84.3)	905 (83.8)	565 (85.2)	
Present	273 (15.7)	175 (16.2)	98 (14.8)	
Missing	60	34	26	
Resection margin, n (%)† ‡				0.54
R0	787 (63.9)	497 (62.9)	290 (65.6)	
R1	187 (15.2)	126 (15.9)	61 (13.8)	
Rx	258 (20.9)	167 (21.1)	91 (20.6)	
Missing	36	26	10	
Treatment, n (%)				<0.001
Endoscopic only	847 (47.0)	529 (47.5)	318 (46.2)	
Primary surgery	535 (29.7)	298 (26.8)	237 (34.4)	
Completion surgery	421 (23.3)	287 (25.7)	134 (19.4)	
ASA, American Society of Anesthesiology; IQR, interquartile range. ^*^P value is derived from descriptive statistics of imputed data. ^†^ The percentage of cases with missing data were: ASA classification, 4.4%; morphology, 1.9%; polyp size, 10.0%; differentiation, 1.1%; lymphovascular invasion, 3.3%; resection margin, 2.0%. All data were available in the remaining categories. Missing data were imputed before analyses were performed. ^‡^ Only patients with an initial local resection.				

## Results

### Patient characteristics and treatment strategies

**Fig. 1:**
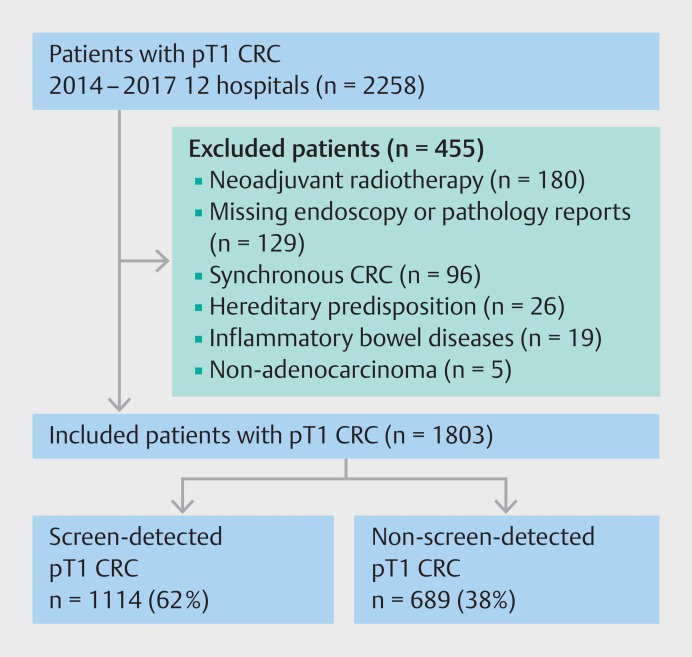
Flowchart of included T1 colorectal cancer (CRC) patients.

A total of 2258 T1 CRC patients were identified in the participating hospitals, of whom 455 were excluded for meeting one or more of the exclusion criteria ( Fig. 1 ). Of the remaining 1803 patients, 1114 (61.8%) were diagnosed through population-based screening and 689 (38.2%) were non-screen detected. In the non-screen-detected group, the indications for the colonoscopy where the T1 CRC was detected were as follows: symptoms, 579/689 (84.0%); incidental finding on imaging, 54/689 (7.8%); surveillance for history of adenomatous polyp(s), 37/689 (5.4%); surveillance for history of CRC (>5 years prior to diagnosis of T1 CRC), 10/689 (1.5%); family history without genetic predisposition, 7/689 (1.0%); reason unknown, 2/689 (0.3%). Screen-detected patients were younger (67 vs. 71 years; *P* < 0.001), more often male (65.2% vs. 57.7%; *P* = 0.002), and had fewer co-morbidities (ASA III/IV 9.3% vs. 20.6%; *P* < 0.001) ( Table 1 ). Left-sided T1 CRCs were more likely to be found in screen-detected patients, while the percentages of right-sided and rectal T1 CRCs were higher in the non-screen-detected patients ( *P* < 0.001). No differences were observed regarding morphology and histopathologic features.

**Fig. 2:**
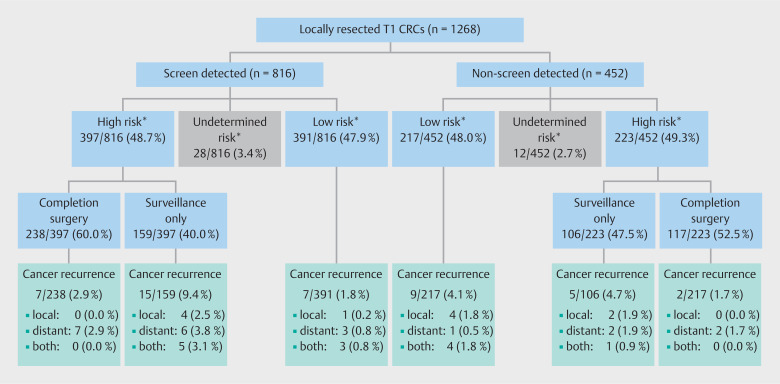
Treatment strategies and oncologic outcomes within the subgroup of 1268 endoscopically resected screen-detected and non-screen-detected T1 colorectal cancers (CRCs), subdivided according to histopathologic risk group. * High risk was defined as having one or more of the following histopathologic risk factors: lymphovascular invasion, poor differentiation, positive resection margin (R1) or non-assessable resection margin (Rx). In the absence of all of these risk factors, a T1 CRC was classified as low risk. When all known risk factors were absent, but one or more risk factors were missing, a T1 CRC was classified as being of undetermined risk.

While the overall surgical resection rate was comparable between the two groups (52.5% and 53.8% in the screen-detected and non-screen-detected groups, respectively), primary surgery was more often performed in non-screen-detected patients (26.8% vs. 34.4%; *P* < 0.001). Of the 1268 patients who were treated with endoscopic resection first, 397/816 screen-detected T1 CRCs (48.7%) exhibited one or more histopathologic high risk features, compared with 223/452 non-screen-detected T1 CRCs (49.3%; *P* = 0.97). The treatment strategy (completion surgery or surveillance) of these patients with endoscopically resected high risk T1 CRCs did not significantly differ between the screen-detected and non-screen-detected patients ( *P* = 0.09) ( Fig. 2 ).

### LNM in surgically treated patients

LNM was detected in 107/956 surgically treated patients (11.2%) ( **Table 1s** ). LNM was more often observed in screen-detected patients compared with non-screen-detected patients (12.6% vs. 8.9%), although the difference in LNM was not significant in univariable analysis (unadjusted odds ratio [OR] 1.49, 95%CI 0.96–2.30). After adjustment for clinical and histopathologic characteristics, detection through screening was not an independent risk factor for LNM (OR 1.41, 95%CI 0.89–2.23). The findings of the sensitivity analysis, which was restricted to patients diagnosed between the ages of 55 and 80, also did not show a significant difference in the risk of LNM between screen-detected and non-screen-detected patients (OR 1.52, 95%CI 0.91–2.53).

### Prediction of LNM

The performance of a model based on a combination of conventional clinical and histologic risk factors for the presence of LNM (age, location, pedunculated vs. nonpedunculated morphology, grade of differentiation, and LVI) was tested in both screen-detected and non-screen-detected surgically treated patients. Calibration curves showed that the calibration of the model was better in the screen-detected T1 CRC group ( **Fig. 1s** ). The AUROCs were not significantly different between screen-detected (0.66, 95%CI 0.59–0.73) and non-screen-detected T1 CRCs (0.60, 95%CI 0.48–0.71; *P* = 0.37), indicating no significant difference in the discriminative ability of the model between the two groups ( **Fig. 2s** ). Finally, a closed testing procedure showed that there was no statistical evidence that the model, which was developed in the total cohort, needed updating in either screen-detected or non-screen-detected patients.

### TTR, MFS, CSS, and overall survival

**Fig. 3:**
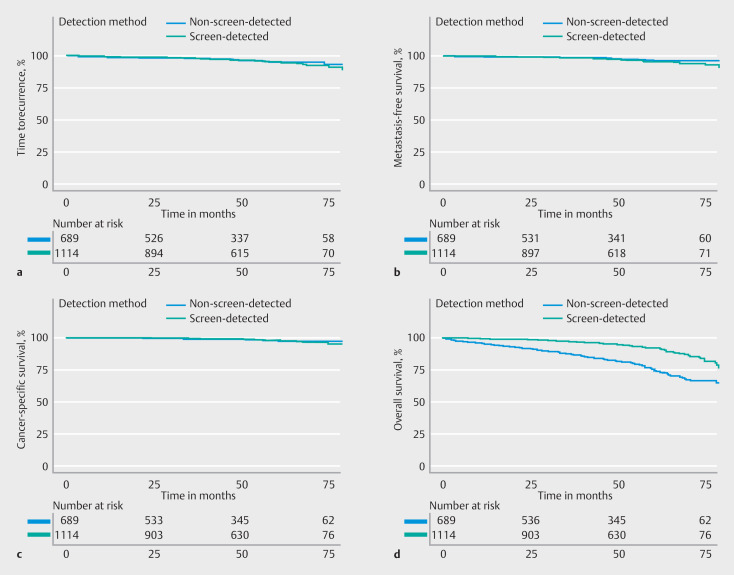
Outcomes of screen-detected and non-screen-detected T1 colorectal cancer (CRC) patients in terms of: **a** time to recurrence; **b** metastasis-free survival; **c** cancer-specific survival; **d** overall survival.

After a median follow-up of 51 months (IQR 30 months), CRC recurrence occurred in 38/1114 screen-detected patients (3.4%) and 24/689 non-screen-detected patients (3.5%) ( **Table 2s** ). Of the screen-detected patients, 19 (1.7%) died of a CRC-related cause: cancer recurrence during follow-up (n = 13); distant metastasis at the time of diagnosis (n = 3); CRC treatment-related cause (n = 3). In the non-screen-detected group, CRC-related death was observed in 11 patients (1.6%), of whom eight died after cancer recurrence and three owing to a CRC treatment-related cause. The estimated 3- and 5-year TTRs among screen-detected T1 CRC patients were similar to those of non-screen-detected patients (adjusted hazard ratio [HR] 1.05, 95%CI 0.62–1.79) ( Fig. 3 **a** ; Table 2 and Table 3 ).

**Table:** **Table 2** Oncologic and survival outcomes of screen-detected and non-screen-detected T1 colorectal cancer (CRC) patients after 3 and 5 years.

	Screen detected (n = 1114)		Non-screen detected (n = 689)		Hazard ratio (95%CI) ^b^	*P* value†
	Event-free* at:		Event-free* at:			
	3 years, % (95%CI)	5 years, % (95%CI)	3 years, % (95%CI) ^a^	5 years, % (95%CI)		
Time to recurrence (TTS)	98.4 (97.6–99.2)	95.4 (93.7–97.0)	98.0 (96.8–99.1)	95.3 (93.3–97.4)	1.05 (0.62–1.78)	0.85
Metastasis-free survival (MFS)	98.7 (98.0–99.4)	95.8 (94.2–97.4)	98.8 (97.9–99.7)	96.7 (94.9–98.5)	1.41 (0.75–2.65)	0.28
Cancer-specific survival (CSS)	99.3 (98.7–99.8)	98.0 (96.9–99.1)	99.0 (98.2–99.8)	97.6 (96.1–99.0)	1.05 (0.47–2.34)	0.91
Overall survival	97.0 (95.9–98.1)	91.9 (89.9–94.0)	87.4 (84.8–90.0)	75.3 (71.4–79.3)	0.51 (0.38–0.68)	<0.001
* Kaplan–Meier method estimates of the percentage of patients without the event of interest at 3 and 5 years, with corresponding 95%CI. ^†^ Hazard ratio and *P* value from multivariable Cox proportional hazards regression model, adjusted for polyp location, treatment strategy, differentiation grade, lymphovascular invasion, and resection margin (for TTR, MFS, and CSS) or age at diagnosis, sex and American Society of Anesthesiology classification (for overall survival).						

**Table:** **Table 3** Multivariable Cox proportional hazard regression analyses for comparison of TTR, MFS, CSS, and overall survival of screen-detected vs. non-screen-detected T1 colorectal cancers.

Covariate, per analysis	Hazard ratio (95%CI)
**Time to recurrence (TTR)**	
Detection method (reference: non-screen detected)	
Screen detected	1.05 (0.62–1.79)
Location (reference: right-sided colon)	
Left-sided colon	0.80 (0.35–1.82)
Rectum	2.09 (0.90–4.79)
Treatment (reference: endoscopic only)	
Primary surgery	1.03 (0.52–2.04)
Completion surgery	0.37 (0.17–0.80)
Differentiation (reference: well/moderate)	
Poor	1.20 (0.42–3.45)
Lymphovascular invasion (reference: absent)	
Present	2.50 (1.35–4.63)
Resection margin (reference: R0)	
R1/Rx	2.12 (1.13–3.94)
**Metastasis-free survival (MFS)**	
Detection method (reference: non-screen-detected)	
Screen-detected	1.41 (0.75–2.65)
Location (reference: right-sided colon)	
Left-sided colon	1.00 (0.37–2.68)
Rectum	2.70 (0.99–7.38)
Treatment (reference: endoscopic only)	
Primary surgery	1.29 (0.58–2.86)
Completion surgery	0.53 (0.23–1.24)
Differentiation (reference: well/moderate)	
Poor	1.50 (0.51–4.43)
Lymphovascular invasion (reference: absent)	
Present	2.67 (1.35–5.25)
Resection margin (reference: R0)	
R1/Rx	2.19 (1.07–4.49)
**Cancer-specific survival (CSS)**	
Detection method (reference: non-screen-detected)	
Screen-detected	1.05 (0.47–2.34)
Location (reference: right-sided colon)	
Left-sided colon	1.03 (0.35–3.02)
Rectum	1.55 (0.47–5.14)
Treatment (reference: endoscopic only)	
Primary surgery	2.87 (0.99–8.29)
Completion surgery	0.49 (0.14–1.67)
Differentiation (reference: well/moderate)	
Poor	2.05 (0.64–6.62)
Lymphovascular invasion (reference: absent)	
Present	3.60 (1.51–8.55)
Resection margin (reference: R0)	
R1/Rx	2.62 (0.85–8.06)
**Overall survival**	
Detection method (reference: non-screen-detected)	
Screen-detected	0.51 (0.38–0.68)
Age at diagnosis	1.07 (1.05–1.09)
Sex (reference: male)	
Female	0.83 (0.63–1.10)
ASA classification (reference: I)	
II	2.86 (1.57– 5.24)
III/IV	5.87 (3.10– 11.10)
ASA, American Society of Anesthesiology.	

Distant metastases were found in 32/38 patients with cancer recurrence in the screen-detected group (84.2%), compared with 15/24 patients in the non-screen-detected group (62.5%). MFS at 3 and 5 years did not differ significantly between the two groups (adjusted HR 1.41, 95%CI 0.75–2.65) ( Fig. 3 **b** ). The estimated 3- and 5-year CSSs were high in both groups and were not significantly different between screen-detected and non-screen-detected patients (adjusted HR 1.05, 95%CI 0.47–2.34) ( Fig. 3 **c** ). Further analyses of oncologic outcomes for the three treatment strategies separately did not show a difference in cancer recurrence or CRC-related death between screen-detected and non-screen-detected T1 CRCs for any of the strategies either ( **Table 3s** ).

As opposed to the cancer-specific outcomes, overall survival was significantly higher in the screen-detected T1 CRC population, with overall survival rates of 97.0% and 91.9% at 3 and 5 years, compared with 87.4% and 75.3% among non-screen-detected T1 CRC patients, even when adjusted for age at diagnosis, sex, and ASA classification (adjusted HR 0.51, 95%CI 0.38–0.68) ( Fig. 3 d).

In the sensitivity analyses, which we restricted to patients aged 55–80 years, still no differences were found in TTR, MFS, and CSS between screen-detected and non-screen-detected T1 CRCs (TTR: adjusted HR 1.08, 95%CI 0.60–1.94; MFS: adjusted HR 1.36, 95%CI 0.69–2.71; CSS: adjusted HR 1.35, 95%CI 0.52–3.49). Overall survival remained significantly higher in the screen-detected T1 CRC patients (3-year overall survival: screen detected 97.0% vs. non-screen detected 88.7%; 5-year overall survival: screen detected 91.9% vs. non-screen detected 79.4%; adjusted HR 0.53, 95%CI 0.39–0.72) ( **Fig. 3s** ).

Within the subgroup of 1268 patients who were treated with endoscopic resection first, oncologic outcomes also did not differ between the screen-detected population and non-screen-detected patients (TTR: adjusted HR 1.02, 95%CI 0.54–1.91; MFS: adjusted HR 1.35, 95%CI 0.63–2.93; CSS: adjusted HR 0.79, 95%CI 0.23–2.69) ( Fig. 2 ). In addition, in this subgroup, overall survival was significantly higher in the screen-detected patients (adjusted HR 0.52, 95%CI 0.37–0.74).

## Discussion

Within this study, we showed that T1 CRCs detected through population-based screening have comparable rates of LNM, and 3- and 5-year TTR, MFS, and CSS compared with non-screen-detected T1 CRCs. In addition, no significant difference in the performance of a prediction model for LNM based on conventional clinical and histologic risk factors was observed between the two groups (AUROC 0.66 vs. 0.60; *P* = 0.47). These findings suggest that the current risk stratification models, based on non-screen-detected T1 CRCs, can be applied to screen-detected T1 CRCs as well. Consequently, there is no need for differential treatment approaches between screen-detected T1 CRCs and non-screen-detected T1 CRCs. However, screen-detected T1 CRC patients had a significantly higher overall survival owing to a lower risk of mortality unrelated to CRC (adjusted HR 0.51, 95%CI 0.38–0.68).

The findings of the present study did not provide support for our hypothesis that there may be disparities in stage distribution and oncologic outcomes between T1 CRCs detected through population-based screening and outside of the screening program. The proportion of LNM did not significantly differ between the two groups, and the observed rates of LNM were consistent with those reported in the literature prior to the implementation of screening programs 11 12 . Distant metastasis at time of diagnosis (stage IV disease) was rare in this cohort, both for screen-detected (0.7%) and non-screen-detected T1 CRCs (0.1%). Therefore, although population-based screening has led to an increased rate of detection of T1 CRCs, the stage distribution of this specific group of patients has not changed. The same holds true for oncologic outcomes during the follow-up period. Within the total cohort, 2.6% of T1 CRCs showed distant metastasis during follow-up, which is in line with previous reports on non-screen-detected T1 CRCs 13 14 15 . Moreover, CSS rates were high for both screen-detected and non-screen-detected T1 CRC patients.

In contrast to the similar oncologic outcomes across the two groups, a significant difference was observed in overall survival, even after adjusting for age at diagnosis, sex, and ASA classification. This difference was mainly explained by a difference in mortality unrelated to CRC. A similar observation was made by de Neree tot Babberich et al. 16 , who observed a significantly lower risk of surgical and non-surgical complications in Dutch screen-detected CRC patients, even after extensive case-mix correction for clinical, surgical, and histopathologic factors. Both the lower non-CRC-related mortality and the lower risk of (non-)surgical complications may be explained by a healthy user bias in the screen-detected population 17 . Factors such as socioeconomic status, ethnicity, educational level, body mass index, and smoking habit were not registered, while there is evidence that these factors vary between screening participants and non-participants 18 19 20 . Therefore, unmeasured confounding in the overall survival analysis cannot be ruled out. Furthermore, patients with a non-screen-detected T1 CRC had an indication to undergo a colonoscopy because of either symptoms, underlying illness, or risk profile, which may have caused a selection bias toward an unhealthier population.

Some limitations need to be taken into account. First, we studied the screen-detected population and the non-screen-detected population in a period during which the screening program was implemented in a stepwise manner by age cohort. This influenced the group composition of screen-detected and non-screen-detected T1 CRC patients in our study; however, we did not find a significant difference in the proportions of LNM between the age cohorts that had not yet received an invitation and those who had been invited to take part in the screening program (data not shown). In addition, preliminary results from the Dutch CRC screening program show that, after two subsequent screening rounds, the stage distribution of screen-detected CRCs remained unchanged 21 . Therefore, we do not believe that the proportion of stage III T1 CRCs at diagnosis in screen-detected and non-screen-detected T1 CRC patients will differ significantly in the current situation where the screening program is fully implemented.

A second limitation is the difference in treatment strategies between screen-detected and non-screen-detected T1 CRCs. As previously observed in a population-based study 1 , screen-detected T1 CRCs were more often treated with local excision first. This may have caused a selection bias toward more high risk cases in the screen-detected primary surgery group. Eventually, however, the same proportion of screen-detected and non-screen-detected patients underwent surgical resection with removal of the draining lymph nodes, as the proportion of completion surgery was higher in the screen-detected group, leading to a similar overall surgical intervention rate. Moreover, screen-detected patients who only underwent local excision showed a similar 5-year TTR and MFS compared with non-screen-detected patients, indicating that both groups seem to have a similar oncologic risk profile.

A post hoc multivariable logistic regression analysis, in which we studied potential factors associated with primary surgery, showed that the difference in treatment strategy may be partly explained by the higher proportion of right-sided tumors found in non-screen-detected patients (data not shown). Local resection options in the proximal colon are limited and sometimes only performed by experienced endoscopists in expert centers. Other factors independently associated with a primary surgical resection were nonpedunculated morphology and larger polyp size. However, this could not completely explain the higher local resection rate in screen-detected patients, because detection through screening showed an independent association with a lower primary surgical resection rate, also after adjustment for ASA classification, age at diagnosis, year of diagnosis, and the aforementioned factors.

It is therefore not completely clear why primary surgery was more often chosen in non-screen-detected patients. Perhaps unmeasured parameters, such as the experience of the endoscopists, differences in optical features, perception of the impact of surgery, and patient preference, may have played an important role. Because a screening endoscopist needs specific accreditation to perform colonoscopies in the Dutch CRC screening program, it might also be that these endoscopists are better equipped to recognize and treat malignant polyps, which could have influenced the treatment strategy.

Finally, although this is a large-sized study, we cannot rule out that an even larger study population would have revealed a significant difference in the proportion of LNM between screen-detected and non-screen-detected patients. The sensitivity analysis, which was restricted to patients diagnosed between the ages of 55 and 80, showed that screen-detected patients had a 1.52 times higher chance of LNM compared with patients outside the screening program, with a confidence interval of 0.91–2.53. It therefore cannot be concluded with complete certainty that screen-detected patients do not have a slightly increased risk of LNM. This potential unrecognized difference in the risk of LNM is however not reflected in a difference in risk of local recurrence and distant metastasis, which are the most important oncologic outcomes. It is therefore more likely that the observed difference in LNM is based on chance or selection, rather than that there is a true clinically relevant difference.

In conclusion, both the rise in T1 CRCs detected through screening and the ongoing shift toward more local resections of early-stage CRC make it important to urgently unravel the oncologic risk profile of screen-detected T1 CRC. Our multicenter study shows that the risks of LNM and cancer recurrence are not significantly different in screen-detected and non-screen-detected T1 CRCs. The comparable performance of conventional risk factors for LNM and the similar rates of TTR, MFS, and CSS observed in the two groups further confirm that T1 CRCs detected through screening can be treated similarly to T1 CRCs detected outside of the screening program.

## Supplementary Material

Supplementary Material Zusatzmaterial
