# Supplementary material for: Oncologic outcomes of screen-detected and non-screen-detected T1 colorectal cancers
Source: Endoscopy. 2024 Mar 13;56(7):484–93. doi: 10.1055/a-2263-2841 (PMC11583000; doi:10.1055/a-2263-2841)
Supplement: Supplementary file 1 — Supplementary Material [file 10-1055-a-2263-2841_22756326.pdf_suppl.pdf.pdf]

Supplementary material

Oncological outcomes of screen-detected and non-screen-detected T1 colorectal cancers

Lisa van der Schee, Krijn J.C. Haasnoot, Sjoerd G. Elias, Kim M. Gijsbers, Yasser A. Alderlieste, Yara Backes, Anne-Marie van Berkel, Femke Boersma, Frank ter Borg, Emilie C.H. Breekveldt, Koen Kessels, Miriam Koopman, Iris Lansdorp-Vogelaar, Monique E. van Leerdam, Gertjan Rasschaert, Ramon-Michel Schreuder, Ruud W.M. Schrauwen, Tom C.J. Seerden, Marcel B.W.M. Spanier, Jochim S. Terhaar Sive Droste, Esther Toes-Zoutendijk, Jurriaan B. Tuynman, Geraldine R. Vink, Wouter H. de Vos tot Nederveen Cappel, Frank P. Vleggaar, Miangela M. Laclé, Leon M. G. Moons

Table of contents **Page**

**Appendix 1s**	Supplementary methods	2
**Table 1s**	Clinical/histopathologic characteristics	3
**Figure 1s**	Calibration plots	5
**Figure 2s**	AUROCs	6
**Table 2s**	Pattern of recurrences	7
**Table 3s**	Oncologic outcomes for different treatment strategies	8
**Figure 3s**	Outcomes for sensitivity analysis	9
**References**		10

van der Schee L et al. Oncologic outcomes of screen-detected and … Endoscopy | © 2024. Thieme. All rights reserved.

Supplementary material

Appendix 1s Supplementary method

Definitions

The physical status of the patients was recorded according to the American Society of Anesthesiology (ASA) classification system. Polyp size, morphology, location, and type of endoscopic treatment were extracted from the endoscopy report. Morphology was defined as either sessile (including flat polyps) or pedunculated. Right-sided location included the cecum, the ascending colon, the transverse colon and the splenic flexure. Left-sided location was defined as the descending colon, the sigmoid and the rectosigmoid. The rectum was recorded separately. Data on histopathological factors were obtained from the pathology report and included differentiation grade (good/moderate vs poor), lymphovascular invasion (LVI) (absent vs present), and resection margin status (negative [R0] vs not assessable [Rx] vs positive [R1]). Submucosal invasion depth was not included in this study, because it is not considered a histopathological risk factor in Dutch guidelines and was therefore poorly reported. Tumor budding, which was not yet incorporated in clinical guidelines and standard histopathological reporting at the time of the study[1–4], could also not be included. Differentiation grade was assessed according to the World Health Organization classification of tumors[5]. Mucinous tumors and signet-ring cell tumors were considered poorly differentiated. Concerning treatment, endoscopic resection only was defined as a local resection without additional surgery, whereas primary surgical resection was considered surgical oncological resection with no prior endoscopic resection. Completion surgery was defined as additional surgical oncological resection after initial endoscopic resection. Transanal minimally invasive surgery and combined endoscopic-laparoscopic surgery were considered endoscopic resections, as no lymphadenectomy is performed in these surgical resections.

van der Schee L et al. Oncologic outcomes of screen-detected and … Endoscopy | © 2024. Thieme. All rights reserved.

Supplementary material

Table 1s. Clinical and histopathological characteristics of 956 surgically treated T1 CRC patients

**Characteristics**	**All surgically treated patients**			**Primary surgery**			**Completion surgery**		
	**Screen-detected T1 CRC** N=585	**Non-screen-detected T1 CRC** N=371	*P* value[a]	**Screen-detected T1 CRC** N=298	**Non-screen-detected T1 CRC** N=237	*P* value[a]	**Screen-detected T1 CRC** N=287	**Non-screen-detected T1 CRC** N=134	*P* value[a]
Age, in years, median (IQR)	67 (8)	69 (15)	0.002	67 (9)	72 (13)	< 0.001	67 (8)	66 (16)	< 0.05
Male sex, n (%)	364 (62.2)	207 (55.2)	0.04	185 (62.1)	128 (54.0)	0.07	179 (62.4)	77 (57.5)	0.34
ASA classification, n (%)			0.07			< 0.01			0.44
I	147 (25.7)	95 (26.7)		82 (27.9)	51 (22.6)		65 (23.4)	44 (33.8)	
II	370 (64.7)	193 (54.2)		182 (61.9)	128 (56.6)		188 (67.6)	65 (50.0)	
III-IV	55 (9.6)	68 (19.1)		30 (10.2)	47 (20.8)		25 (9.0)	21 (16.2)	
missing	13	15		4	11		9	4	
Location, n (%)			< 0.001			0.17			< 0.05
Right-sided colon	147 (25.1)	113 (30.5)		113 (37.9)	89 (37.5)		34 (11.8)	24 (17.9)	
Left-sided colon	371 (63.4)	189 (50.9)		142 (47.7)	100 (42.2)		229 (79.8)	89 (66.4)	
Rectum	67 (11.5)	69 (18.6)		43 (14.4)	48 (20.3)		24 (8.4)	21 (15.7)	
Morphology, n (%)			0.49			0.71			0.55
Non-pedunculated	430 (75.3)	279 (77.5)		258 (90.8)	204 (89.5)		172 (59.9)	75 (56.8)	
Pedunculated	141 (24.7)	81 (22.5)		26 (9.2)	24 (10.5)		115 (40.1)	57 (43.2)	
missing	14	11		14	9		0	2	
Diameter of polyp, in mm, median (IQR)	20 (15)	25 (20)	< 0.001	25 (10)	30 (20)	0.11	15 (13)	20 (15)	< 0.05
missing	66	74		51	64		3	4	
Differentiation, n (%)			1.00			0.69			0.38
Well/moderate	539 (92.8)	344 (93.0)		282 (94.6)	226 (95.8)		257 (90.8)	118 (88.1)	
Poor/signet ring cell	42 (7.2)	26 (7.0)		16 (5.4)	10 (4.2)		26 (9.2)	16 (11.9)	
missing	4	1		0	1		4	0	
Lymphovascular invasion, n (%)			0.04			0.04			0.63
Absent	431 (76.0)	297 (82.0)		262 (88.5)	221 (94.0)		169 (62.4)	76 (59.8)	
Present	136 (24.0)	65 (18.0)		34 (11.6)	14 (6.0)		102 (37.6)	51 (40.2)	
missing	18	9		2	2		16	7	
Resection margin, n (%)[b]			0.67						0.67
R0	103 (37.6)	50 (38.2)		-	-	-	103 (37.6)	50 (38.2)	
R1	82 (29.9)	34 (25.9)					82 (29.9)	34 (25.9)	
Rx	89 (32.5)	47 (35.9)					89 (32.5)	47 (35.9)	
missing	13	3					13	3	

 © 2024. Thieme. All rights reserved.

Supplementary material

Treatment, n (%)<br>Primary surgery<br>Completion surgery	 298 (50.9)<br>287 (49.1)	 237 (63.9)<br>134 (36.1)	< 0.001	-	-	-	-	-	-
Lymph nodes retrieved, median (IQR)	14 (7)	14 (8)	0.36	15 (10)	15 (8)	0.90	13 (7)	13 (7)	0.77
LNM, n (%)	74 (12.6)	33 (8.9)	0.07	39 (13.1)	15 (6.3)	< 0.05	35 (12.2)	18 (13.4)	0.84

ASA = American Society of Anaesthesiology; CRC = colorectal cancer; IQR = interquartile range; LNM = lymph node metastasis

a= p-value is derived from descriptive statistics of imputed data

b= only patients with completion surgery were included

van der Schee L et al. Oncologic outcomes of screen-detected and … Endoscopy | © 2024. Thieme. All rights reserved.

Supplementary material

Figure 1s. Calibration plots of the logistic regression model with conventional risk factors for lymph node metastasis (LNM) show that the predicted risks are more accurate in the screen-detected group of T1 CRCs. For higher predicted risks, the model tends to underestimate the risk of LNM in both screen- and non-screen-detected T1 CRCs. (A) Calibration plot for the total cohort. (B) Calibration plot for the group of screen-detected T1 CRCs. (C) Calibration plot for the group of non-screen-detected T1 CRCs.

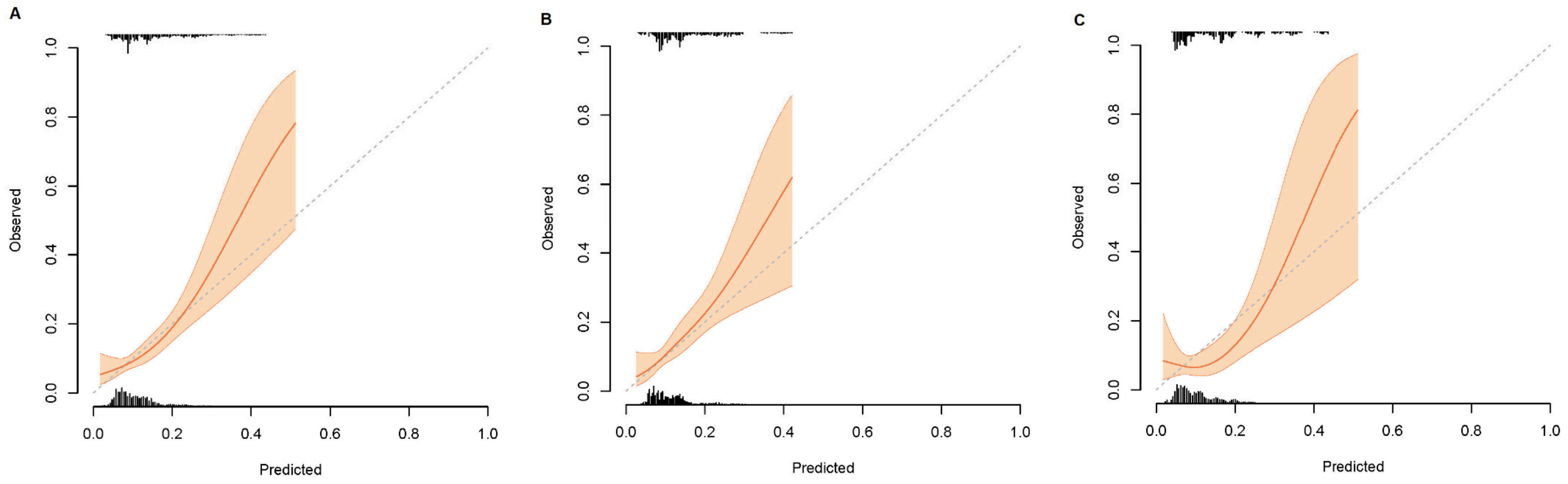

© 2024. Thieme. All rights reserved.

Supplementary material

Figure 2s. Area under the receiver operating curve (AUC) for lymph node metastasis (LNM) in screen-detected and non-screen-detected T1 CRCs based on the logistic regression model with conventional risk factors for LNM.

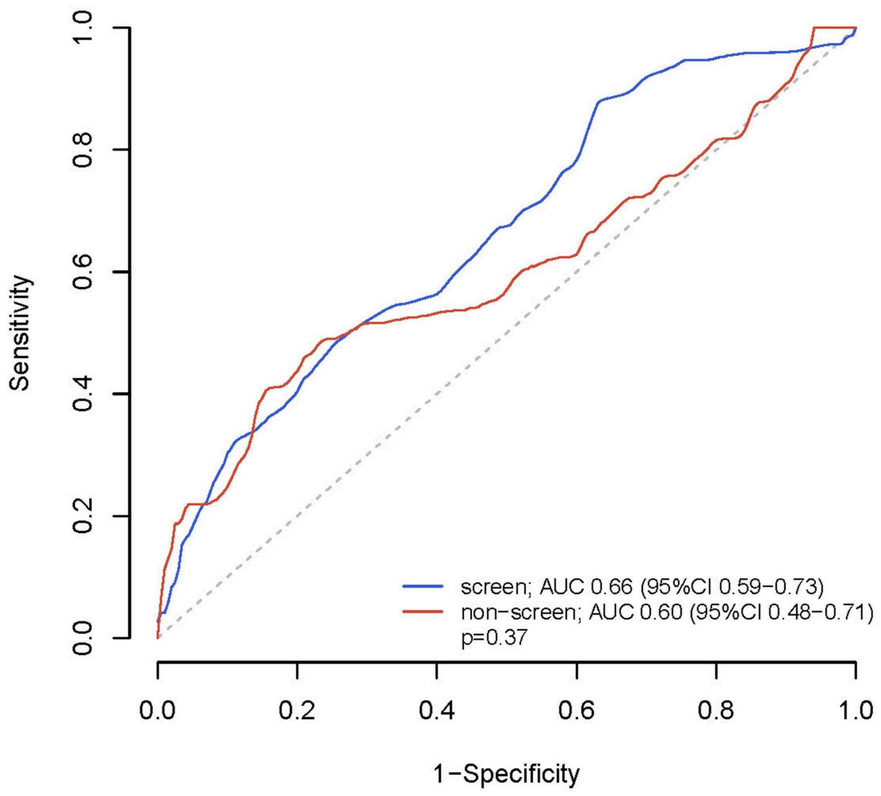

van der Schee L et al. Oncologic outcomes of screen-detected and … Endoscopy | © 2024. Thieme. All rights reserved.

Supplementary material

Table 2s. Pattern of recurrences of screen-detected and non-screen-detected T1 CRCs, stratified according to initial T1 CRC location

Location of recurrence	Recurrences of screen-detected T1 CRC N = 38		Recurrences of non-screen-detected T1 CRC N = 24	
	**Colon** N = 17	**Rectum** N = 21	**Colon** N = 14	**Rectum** N = 10
Local recurrence[a]	6	10	8	7
Distant recurrence				
Liver	8	3	2	1
Lung	2	3	3	1
Lymph node(s)[b]		3		3
Bone	1			
Peritoneum		1	1	
Elsewhere in the bowel		1		
Liver + bone				1
Liver + lung		1		
Lung + lymph node(s)	2	2		
Liver + lymph node(s)	1	1		
Liver + lung + lymph node(s)	1	1	2	
Liver + lung + brain		1		
Bone + vaginal + bladder + lymph node				1

CRC = colorectal cancer
[a]Ten screen-detected and six non-screen-detected patients had both a local and distant recurrence
[b]Only lymph node metastases outside the initial resection area were considered distant recurrences

van der Schee L et al. Oncologic outcomes of screen-detected and … Endoscopy | © 2024. Thieme. All rights reserved.

Supplementary material

Table 3s. CRC-related oncological outcomes during follow-up of all 1803 T1 CRC patients in total and in subgroups according to treatment strategy

	All patients		Endoscopic resection only		Primary surgery		Completion surgery	
	**Screen-detected T1 CRC** N= 1114	**Non-screen-detected T1 CRC** N= 689	**Screen-detected T1 CRC** N= 529	**Non-screen-detected T1 CRC** N= 318	**Screen-detected T1 CRC** N= 298	**Non-screen-detected T1 CRC** N= 237	**Screen-detected T1 CRC** N= 287	**Non-screen-detected T1 CRC** N= 134
**Recurrences, total**	38 (3.4%)	24 (3.5%)	21 (4.0%)	14 (4.4%)	9 (3.0%)	8 (3.4%)	8 (2.8%)	2 (1.5%)
Local recurrence	6 (0.5%)	9 (1.3%)	5 (0.9%)	6 (1.9%)	1 (0.3%)	3 (1.3%)	0 (0.0%)	0 (0.0%)
Distant recurrence	22 (2.0%)	9 (1.3%)	8 (1.5%)	3 (0.9%)	6 (2.0%)	4 (1.7%)	8 (2.8%)	2 (1.5%)
Local + distant recurrence	10 (0.9%)	6 (0.9%)	8 (1.5%)	5 (1.6%)	2 (0.7%)	1 (0.4%)	0 (0.0%)	0 (0.0%)
**Distant metastasis at baseline**	8 (0.7%)	1 (0.1%)	2 (0.4%)	0 (0.0%)	3 (1.0%)	0 (0.0%)	3 (1.0%)	1 (0.7%)
**CRC-related death**	19 (1.7%)	11 (1.6%)	6 (1.1%)	4 (1.3%)	9 (3.0%)	5 (2.1%)	4 (1.4%)	2 (1.5%)
Death due to CRC progression	16 (1.4%)	8 (1.2%)	6 (1.1%)	3 (0.9%)	6 (2.0%)	3 (1.3%)	4 (1.4%)	2 (1.5%)
CRC treatment-related death	3 (0.3%)	3 (0.4%)	0 (0.0%)	1 (0.3%)	3 (1.0%)	2 (0.8%)	0 (0.0%)	0 (0.0%)

CRC = colorectal cancer

 | © 2024. Thieme. All rights reserved.

Supplementary material

Figure 3s. Outcomes of screen-detected and non-screen-detected T1 colorectal cancer (CRC) patients in a subgroup of patients aged 55-80 years. (A) Time to recurrence (TTR). (B) Metastasis-free survival (MFS). (C) Cancer-specific survival (CSS). (D) Overall survival (OS).

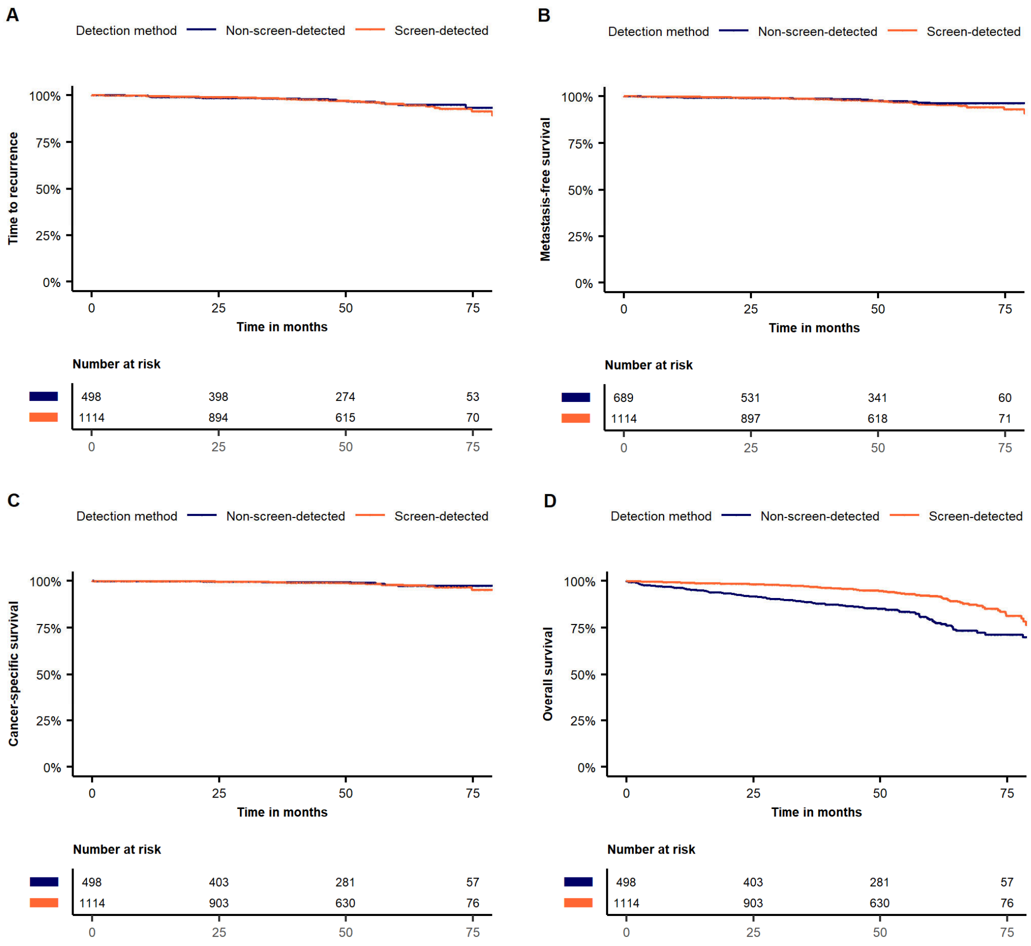

 © 2024. Thieme. All rights reserved.

Supplementary material

References

[1] Ferlitsch M, Moss A, Hassan C, et al. Colorectal polypectomy and endoscopic mucosal resection (EMR): European Society of Gastrointestinal Endoscopy (ESGE) Clinical Guideline. Endoscopy 2017; 49: 270–297. doi:10.1055/s-0043-102569

[2] Hashiguchi Y, Muro K, Saito Y, et al. Japanese Society for Cancer of the Colon and Rectum (JSCCR) guidelines 2019 for the treatment of colorectal cancer. Springer Singapore; 2020

[3] Richtlijn Colorectaal carcinoom (CRC) versie 4.0. 2019

[4] Rosty C, Brown I, Cooper H, et al. Colorectal Excisional Biopsy (Polypectomy) Histopathology Reporting Guide. 2020

[5] Bosman FT, Carneiro F, Hruban RH, et al. WHO Classification of Tumours of the Digestive System. 4th Aufl. Geneva: WHO, IARC; 2010

van der Schee L et al. Oncologic outcomes of screen-detected and … Endoscopy | © 2024. Thieme. All rights reserved.
